# Learning tactile skills through curious exploration

**DOI:** 10.3389/fnbot.2012.00006

**Published:** 2012-07-23

**Authors:** Leo Pape, Calogero M. Oddo, Marco Controzzi, Christian Cipriani, Alexander Förster, Maria C. Carrozza, Jürgen Schmidhuber

**Affiliations:** ^1^Istituto Dalle Molle di Studi sull'Intelligenza Artificiale, Università della Svizzera ItalianaLugano, Switzerland; ^2^The BioRobotics Institute, Scuola Superiore Sant'AnnaPisa, Italy

**Keywords:** active learning, biomimetic robotics, curiosity, intrinsic motivation, reinforcement learning, skill learning, tactile sensing

## Abstract

We present curiosity-driven, autonomous acquisition of tactile exploratory skills on a biomimetic robot finger equipped with an array of microelectromechanical touch sensors. Instead of building tailored algorithms for solving a specific tactile task, we employ a more general curiosity-driven reinforcement learning approach that autonomously learns a set of motor skills in absence of an explicit teacher signal. In this approach, the acquisition of skills is driven by the information content of the sensory input signals relative to a learner that aims at representing sensory inputs using fewer and fewer computational resources. We show that, from initially random exploration of its environment, the robotic system autonomously develops a small set of basic motor skills that lead to different kinds of tactile input. Next, the system learns how to exploit the learned motor skills to solve supervised texture classification tasks. Our approach demonstrates the feasibility of autonomous acquisition of tactile skills on physical robotic platforms through curiosity-driven reinforcement learning, overcomes typical difficulties of engineered solutions for active tactile exploration and underactuated control, and provides a basis for studying developmental learning through intrinsic motivation in robots.

## 1. Introduction

Complex robots typically require dedicated teams of control engineers that program the robot to execute specific tasks in restricted laboratory settings or other controlled environments. Slight changes in the task requirements or the robot's environment often require extensive re-programming, calibration, and testing to adjust the robot to the changed conditions. The implementation of these tasks could be sped up significantly if the robot autonomously develops and maintains some knowledge about its own capabilities and the structure of the environment in which it lives. Instead of placing the task of supplying the robot with such knowledge in the hands of the robot's creator, *curious* robots actively explore their own capabilities and the structure of their environment even *without an externally specified goal*. The structure found in the environment and its relation to the robot's own actions during curious exploration could be stored and used later to rapidly solve externally-specified tasks.

A formalization of the idea of curious exploratory behavior is found in the work of Schmidhuber (2010) and references therein. The theory of intrinsically-motivated learning developed in these works considers active machine *learning agents* that try to become more efficient in storing and predicting the observations that follow from their actions. A major realization of Schmidhuber (2010), is that curious behavior should not direct the agent toward just any unknown or unexplored part of its environment, but to those parts where it expects to *learn* additional patterns or regularities. To this end, the learning agent should keep track of its past learning *progress*, and find the relation between this progress and its own behavior. Learned behaviors that lead to certain regular or predictable sensory outcomes, can be stored in the form of *skills*. Bootstrapping the skills learned in this fashion, the agent can discover novel parts of the environment, learn composite complex skills, and quickly find solutions to externally-specified tasks.

This work presents curiosity-driven, autonomous acquisition of tactile exploratory skills on a biomimetic robot finger equipped with an array of microelectromechanical touch sensors. We show that from active, curiosity-driven exploration of its environment, the robotic system autonomously develops a small set of basic motor skills that lead to different kinds of tactile input. Next, the system learns how to exploit the learned motor skills to solve supervised texture classification tasks. Our approach demonstrates the feasibility of autonomous acquisition of tactile skills on physical robotic platforms through curiosity-driven reinforcement learning, overcomes typical difficulties of engineered solutions for tactile exploration and underactuated control, and provides a basis for studying curiosity-driven developmental learning in robots.

Since both theory and practically-feasible algorithms for curiosity-driven machine learning have been developed only recently, few robotic applications of the curiosity-driven approach have been described in the literature thus far. Initial robotic implementations involving vision-based object-interaction tasks are presented in Kompella et al. (2011). A similar approach has been described by Gordon and Ahissar (2011) for a simulated whisking robot. Examples of alternative approaches to curiosity-driven learning on simple robots or simulators can be found in the work of Oudeyer et al. (2007); Vigorito and Barto (2010); Konidaris et al. (2011); Mugan and Kuipers (2012). None of these works consider curiosity-driven development of tactile skills from active tactile exploration.

The rest of this work is organized as follows: section 2 presents the curiosity-driven learning algorithm and the tactile robotic platform used in the experiments. Section 3.1 illustrates the operation of the curiosity-driven reinforcement learning algorithm on a simple toy-problem. The machine learning approach for tactile skill learning presented here has not been published before, and will be described and compared to other approaches where relevant for tactile skill learning. Section 3.2 then shows the learning of basic tactile skills on the robotic platform and how these can be exploited in an externally-specified surface classification task. Section 4 discusses the results and the relevance of active learning in active tactile sensing.

## 2. Materials and methods

### 2.1. Curiosity-driven modular reinforcement learning

#### 2.1.1. Skill learning

The learning of tactile skills is done here within the framework of reinforcement learning (e.g., Kaelbling et al., 1996). A reinforcement learner (RL) addresses the problem which *actions* to take in which *states* in order to maximize its cumulative expected *reward*. The RL is not explicitly taught which actions to take, as in supervised machine learning, but must instead explore the environment to discover which actions yield the most cumulative reward. This might involve taking actions that yield less immediate reward than other actions, but lead to higher reward in the long-term. When using RLs for robot control, states are typically abstract representations of the robot's sensory inputs, actions drive the robot's actuators, and the rewards represent the desirability of the robot's behavior in particular situations. Learning different skills here is done with a *modular* reinforcement learning architecture in which each module has its own reward mechanism, and when executed, produces its own behavior.

Most modular reinforcement learning approaches address the question how to split up a particular learning task into subtasks each of which can be learned more easily by a separate module. In the curiosity-driven learning framework presented here, there is no externally-specified task that needs to be solved or divided. Instead, the modules should learn different behaviors based on the structure they discover in the agent's sensory inputs. This is done by reinforcement learning modules that learn behaviors that lead to particular kinds of sensory inputs or events, and then terminate. The different *kinds* of sensory events are distinguished by another module, which we here call an abstractor. An abstractor can be any learning algorithm that learns to represent the structure underlying its inputs into a few relevant components. This could for example be an adaptive clustering method, an autoencoder (e.g., Bourlard and Kamp, 1988), qualitative state representation (Mugan and Kuipers, 2012) or (including the time domain) a slow-feature analysis (Kompella et al., 2011). Each component of the abstractor is coupled to a RL module that tries to generate *stable* behaviors that lead to sensory inputs with the coupled abstractor state, and then terminates. The resulting modules learn the relation between the part of their sensory inputs that can be directly affected through their own actions, and the abstract structure of their sensory inputs. In other words, the system learns different *skills* that specify *what* sensory events can occur, and *how* to achieve those events. As the behaviors learned by these modules depend on the ability of the system to extract the structure in its sensory input, and not on some externally-provided feedback, we call these modules intrinsically-rewarded skills (inSkills).

Apart from inSkills, we also use externally-rewarded skills (exSkills) that are learned through external reward from the environment, and a small number of other modules whose operation will be detailed below. Modules can take two kinds of actions (1) execute a *primitive* that translates directly into an actuator command and (2) execute another module. When the executed module collects sensory inputs with its corresponding abstractor state, it terminates and returns control to the calling module. The possibility of executing another module as part of a skill allows for cumulative learning of more complex, composite skills. In the initial learning stages, there is not much benefit in selecting another module as an action, as most modules have not yet developed behaviors that reliably lead to different sensory events. However, once the modules become specialized, they may become part of the policy of another module. To prevent modules from calling themselves directly, or indirectly via another module, the RL controller keeps track of the selected modules on a calling stack, and removes the currently executing module and its caller modules from the available action set. In this fashion, only modules that are not already on the calling stack can be selected for execution.

It is not uncommon for modular architectures to instantiate additional modules during the learning process. This comes at a disadvantage of specifying and tuning *ad-hoc* criteria for module addition and pruning. Instead, we use a learning system with a fixed number of modules, which has to figure out how to assign those modules to the task at hand. Although this system is by definition limited (but so is any physical system), flexibility, and cumulative learning are achieved through the hierarchical combination of modules; once the system acquires a new skill, it could use that skill as part of another skill to perform more complex behaviors.

During curious exploration of its environment, the learning agent is driven by a module that tries to improve the reliability of the inSkill behaviors. The idea of using the learning progress of the agent as reward is closely following the work of Schmidhuber (2010). However, the focus here is not so much on the ability of the abstractor predict or compress any observations, but on finding stable divisions of sensory inputs produced by the agent's behavior into a few components. The intrinsic reward is not just the learning progress of the abstractor, but also includes the improvement in the RL's ability to produce the different sensory events distinguished by the abstractor. In essence, the role of learning progress is taken over here by stability progress, which involves the distribution of the agent's limited computational and physical resources such that the most relevant (relative to the system's learning capabilities) sensory events can be reliably produced. This strong relation between distinct sensor abstractions and the ability to learn behaviors that lead to those abstractions has also been argued for by Mugan and Kuipers (2012).

#### 2.1.2. Adaptive model-based reinforcement learning

Although the abstractors and RLs could in principle be instantiated with a range of different machine learning methods, in practice, especially in robotic practice, few algorithms can be successfully used. The main challenges for curiosity-driven learning on actual hardware are: (1) the algorithms have to learn from much smaller amounts of samples (in the order of 10^2^–10^3^) than are typically assumed to be available in the machine learning literature (often more than 10^4^ to solve even the simplest tasks); (2) typical machine learning approaches assume that training samples are generated from a stationary distribution, while the whole purpose of curiosity-driven learning is to make novel parts of the environment and action space available to the robot *during* and *as a result of* learning; (3) typical reinforcement learning algorithms assume a stationary distribution of the reward, while the intrinsic reward signal in curiosity-driven learning actually decreases as a result of learning the behavior that leads to this reward. None of these challenges is considered solved in the area of practical machine learning; mathematically optimal universal ways of solving them (Schmidhuber, 2006) are not practical. In the present work, we employ various machine learning techniques that have been proposed before in the literature, and introduce some new approaches we are not aware of having been described before. The main criterion for choosing the techniques described below was not their theoretical elegance, efficiency, or even optimality, but their robustness to the challenges addressed above.

To learn effectively from the small amount of samples that can be collected from the robotic platform, the learning system trains a Markov model from the collected data, and generates training data for the reinforcement learning algorithm from this model. A Markov model represents the possible states S of its environment as a set of numbers s ∈ S. In each state, a number of actions a ∈ A are available that lead to (other) states $s^{\prime }\;\in \;S$ with probability *p*(*s*′|*s*, *a*). While a primitive action takes one timestep, skills taking several timesteps might also be selected as actions. The model therefore also stores the duration *d*(*s*, *a*, *s*′) of an action in terms of the number of primitive actions.

The Markov model is further augmented to facilitate learning during the dynamic expansion of the agent's skills and exploration of the environment. For each module ${ℳ}_{j}$ and each transition (*s*, *a*, *s*′), the model keeps track of: (1) the short-term reward *r*_*j*_(*s*, *a*, *s*′) provided by the module's reward system; (2) the probability *z*_*j*_(*s*, *a*, *s*′) of terminating the module's policy; (3) the long-term reward *q*_*j*_(*s*, *a*, *s*′) that changes on a slower timescale than the short-term reward. Instead of accumulating the Markov model's learned values over the whole learning history, all model values are updated with a rule that gives more weight to recently-observed values and slowly forgets observations that happened a long time ago:

(1) $$m(s,a,s^{\prime })\leftarrow (1-w_{*})m(s,a,s^{\prime })+w_{*}v(s,a,s^{\prime }),$$

with model values *m* = {*d*, *q*, *r*, *z*}, update weights w_*_ = {*w*_*d*_, *w*_*q*_, *w*_*r*_, *w*_*z*_}, and observed values *v* = {*d*, *q*, *r*, *z*}. The short-term rewards, termination probabilities, and transition durations are updated according to Equation 1 for every observation, while the long-term reward is updated for all *q*(*s*, *a*, *s*′) after processing of a number of samples equal to the reinforcement learning episode length. Transition probabilities are updated by adding a small constant *w*_*p*_ to *p*(*s*′|*s*, *a*), and then rescaled such that $\sum {}_{s^{\prime }\in S}p(s^{\prime }|s,a)=1$. As the model values adjust to the changing skills, previously learned transitions become less likely. For efficiency reasons we prune model values *d*, *p*, *r*, *z* for which the transition probabilities have become very small (*p*(*s*′|*s*, *a*) < *w*_*o*_) after each model update. Together, these update rules ensure that the agent keeps adapting to newly acquired skills and changing dynamics of its expanding environment. Increasing (decreasing) the model parameters {*w*_*d*_, *w*_*o*_, *w*_*q*_, *w*_*r*_, *w*_*z*_} leads to the development of more flexible (more stable) behaviors.

The values stored in the Markov model for each module are used by reinforcement learners to learn policies that maximize the cumulative module rewards. The RLs keep track of how much each state-action pair (*s*, *a*) contributes to the cumulative reward *r* when following the current action-selection policy. In reinforcement learning these state-action values are known as a *Q*-values:

(2) $$Q(s,a)=\sum_{t=0}^{\infty }\gamma^{t}r(t),$$

where γ is a discount factor that weights the importance of immediate versus future rewards, and *t* is time. The RL selects actions *a* in state *s* according its current policy π(*s*):

(3) $$\pi (s)=\arg\underset{a\in A}{\max}Q^{\pi }(s,\;a).$$

An efficient algorithm for learning those *Q*-values is least-squares policy iteration (LSPI; Lagoudakis and Parr (2003)). LSPI represents the estimated *Q*-values as an ω-weighted linear combination of κ features of state-action pairs ɸ(*s*, *a*):

(4) $$\hat{Q}(s,a)=\sum_{j=1}^{\kappa }{ɸ}_{j}(s,a)\omega_{j},$$

where ω_*j*_ are the parameters learned by the algorithm. The feature function ɸ(*s*, *a*) used here represents state-action pairs as binary feature vectors of length κ = |S||A|, with a 1 at the index of the corresponding state-action pair, and a 0 at all other indices. LSPI sweeps through a set of *n* samples *D* = {(*s*_*i*_, *a*_*i*_, *s*′_*i*_, *r*_*i*_, *p*(*s*′_*i*_|*s*_*i*_, *a*_*i*_)) | *i* = 1,…,*n*} generated from the model, and updates its estimates of parameter vector ω as:

(5) $$\omega =A^{-1}b$$

(6) $$A=\sum_{i=1}^{n}\left[ɸ(s_{i},a_{i}){\left(ɸ(s_{i},a_{i})-\sum_{s^{\prime }\in D}\gamma p(s^{\prime }|s_{i},a_{i})ɸ(s^{\prime },\pi (s^{\prime }))\right)}^{T}\right]$$

(7) $$b=\sum_{i=1}^{n}\left[ɸ(s_{i},a_{i})r_{i}\right].$$

Because actions with different durations are possible in our implementation, we slightly alter Equations 6 and 7 to take into account the duration *d*_*i*_ of a transition, both in the discount factor γ and the module reward *r*_*i*_:

(8) $$A=\sum_{i=1}^{n}\left[ɸ(s_{i},a_{i}){\left(ɸ(s_{i},a_{i})-\sum_{s^{\prime }\in D}\gamma^{d_{i}}p(s^{\prime }|s_{i},a_{i})ɸ(s^{\prime },\pi (s^{\prime }))\right)}^{T}\right]$$

(9) $$b=\sum_{i=1}^{n}\left[ɸ(s_{i},a_{i})\gamma^{d_{i}-1}r_{i}\right].$$

#### 2.1.3. Skill types

The curiosity-driven learning agent uses four different types of reinforcement learning modules:

An **explorer** module is a naive curiosity module that tries to find novel observations around previous novel observations, but does not exploit any further structure of the environment. The reward mechanism of an explorer uses the Markov model to keep track of the number of times a certain state was visited, and rewards transitions (*s*, *a*, *s*′) inverse-proportionally to the number of times *s*′ was visited: *r*_*j*_(*s*, *a*, *s*′) = *e*^−*c*(·,·,*s*′)^, where *e* is the natural logarithm base, and *c*(·,·,*s*′) the number of times a transition led to *s*′. This leads to a policy that drives the agent toward yet unexplored parts of the environment, thus speeding up initial exploration.

**InSkill** modules exploit regularities in the environment to learn behaviors that lead to particular kinds of sensory events. Sensory inputs are grouped in an unsupervised manner by the abstractor into separate abstractor states *y*_*j*_. The behavior that leads to each abstractor state *y*_*j*_ is learned by an individual reinforcement learning module ${ℳ}_{j}$. The reward mechanism of these RLs reflects the reliability with which a transition leads to a particular abstractor state. A reward of 1 is given when transition (*s*, *a*, *s*′) leads to *y*_*j*_, and a reward of 0 otherwise. In combination with the update rule in Equation 1, this yields high model reward values *r*_*j*_(*s*, *a*, *s*′) for transitions that reliably lead to the corresponding abstractor state *y*_*j*_, and low model reward values otherwise. An inSkill terminates when a transition produces its coupled abstractor state. When a module terminates because of other reasons (e.g., reaching the maximum number of allowed timesteps), a failure reward −*w*_*f*_ (i.e., penalty) is added to *r*_*j*_(*s*, *a*, ·). The reason this penalty is given to all *s*′ ∈ *S*, is that it is unknown to which state the transition would have led if the module had terminated successfully.

Note that no direct feedback exists between the ability of the abstractor to separate sensory events, and the ability of the RLs to learn behaviors that leads to those events (as is done in some RL approaches). However, there is a behavioral feedback in the sense that the total learning system favors behaviors that lead to those sensory events that can *reliably* be distinguished by the abstractor.

The skill **progressor** drives the overall behavior of the agent when running in curious exploration mode. The progressor executes those skills that are (re)adapting their expertise. Both increase and decrease in the long-term reward collected by a skill implies it is adjusting to a more stable policy, so the progressor is rewarded for the absolute *change* in long-term reward of the inSkills:

(10) $$r_{i}(\cdot ,a_{i},\cdot )=\sum_{\left(s,a,s^{\prime })\in (S\times A\times S\right)}\text{abs}\left(p(s^{\prime }|s,a)\Delta q_{i}(s,a,s^{\prime })\right).$$

and uses a fixed reward *r*_*x*_ for explorer modules.

An **exSkill** module learns to maximize externally-provided reward. Just as the other skills, exSkills can choose to execute other skills, thus exploiting skills that have been learned through the intrinsic reward system. Reward is given for reaching a designer-specified goal, which then also terminates the module.

Apart from the termination condition mentioned in the above description, all modules also terminate after a fixed maximum number of actions τ_*z*_.

All RL modules simultaneously learn from all samples (off-policy). However, modules that execute other modules as part of their own policy, learn about the actual behavior (on-policy) of the executed modules. While off-policy learning facilitates rapid learning of all modules in parallel, it also changes a module's behavior without its explicit execution, leading to potentially incorrect policies in modules that select the changed modules as part of their own policy. This issue is resolved as a side-effect of using a progressor, which is rewarded for, and executes the changing modules, leading to additional sampling of the changed modules until they stabilize.

Apart from the exploration done by the explorer module, a fixed amount of exploration is performed in each module by selecting an untried action from the available action set with probability ∈ instead of the action with the maximum *Q*-value. In case no untried actions are available for exploration, an action is selected with uniform probability from the available action set. Such a policy is called ∈-greedy.

### 2.2. Robotic platform for tactile skill learning

**Figure 1:**
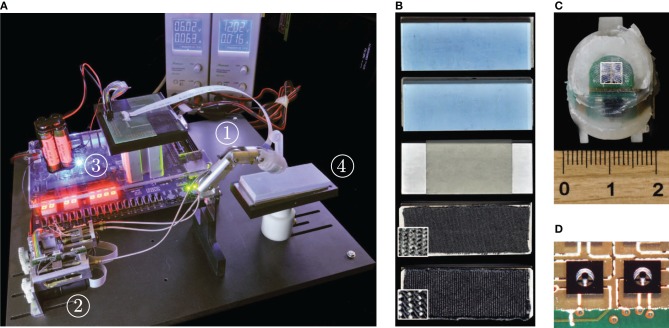
**Pictures of the experimental setup. (A)** Tactile platform with (1) the robotic finger, (2) actuator modules, (3) sensor processing facilities, and (4) housing for replaceable surface blocks. **(B)** Surfaces used in tactile skill learning experiments. From top to bottom: two regular-grated plastic surfaces with 320 and 480 μm spacings, paper, and two denim textiles. The highlighted areas show enlargements of 2 × 2 mm areas. **(C)** Fingertip with a 2 × 2 tactile sensor array in the highlighted area, covered with finger-printed packaging material. The ruler shows the scale in cm. **(D)** Closeup of 2 MEMS tactile sensor units.

We use the curiosity-driven machine learning framework to investigate curiosity-driven learning of tactile skills on a robotic platform specifically designed for active tactile exploration. The platform (Figure 1A) consists of a robotic finger with a tactile sensor in its fingertip, actuation and processing units, and a housing for replaceable blocks with different surfaces. The details of each of those components are given in the remainder of this section.

#### 2.2.1. Biomimetic robotic finger

**Figure 2:**
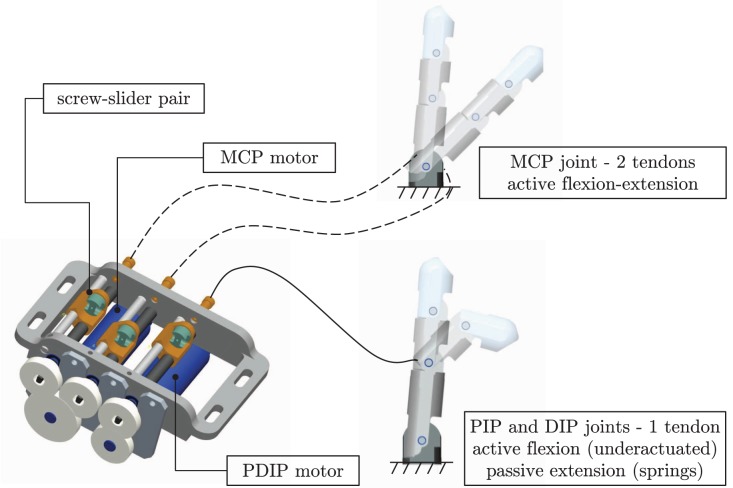
**Robotic finger actuation; MCP and combined PIP and DIP (PDIP) are shown separately**.

The human-sized (Buchholz et al., 1992) biomimetic robotic finger used in the active learning experiments is composed of three phalanxes and three flexion joints: a metacarpophalangeal (MCP) joint, a proximal interphalangeal (PIP) joint, and a distal interphalangeal (DIP) joint (see Figure 2). Unlike the natural finger, no abduction of the MCP joint is possible, since the task under investigation (i.e., an exploratory trajectory) requires the fingertip to move in two dimensions only. Like the natural finger, the robotic finger is driven by tendons and underactuated; the three joints are actuated by just two motors. Underactuation reduces design complexity and allows self-adaptation and anthropomorphic movements similar to human exploratory tasks.

The finger is driven by two direct-current (DC) motors (model 1727, Faulhaber Minimotor; gear head ratio 14:1). One motor actively actuates the flexion and extension of the MCP joint by means of two lead screw pairs with opposite screw handedness (agonist-antagonist action, Figure 2). The second motor actuates the flexion of the PIP and DIP underactuated pair (PDIP hereafter) by pulling the tendon. Extension of the PDIP joints during tendon release is achieved passively through torsional springs housed inside the joints. The DC-motors are integrated with optical encoders that monitor the released tendon-length, enabling position control in motor space. Additionally, tension sensors are integrated in the tendons. Each motor is controlled by a low-level motion controller implementing position, tendon tension, torque (motor current) control, and monitoring. The low-level motion controllers are directly controllable by a host PC through a RS232 serial communication bus.

Due to the underactuated architecture and absence of joint-angle sensors, the kinematics of the finger are not unique and can only be solved by considering the dynamics of the robot and its interaction with the environment. Control and monitoring in motor space does not allow for unique control and monitoring in fingertip space, unless the full dynamic model of the finger and its interaction with the touched surface is computed. This makes it difficult to control the finger by means of conventional control strategies (Arai and Tachi, 1991).

#### 2.2.2. Fingertip with MEMS tactile sensor array

The tip of the robotic finger holds a 2 × 2 array of 3D microelectromechanical system (MEMS) tactile sensors (see also Oddo et al., 2011b) created with silicon microstructuring technologies. Each 1.4 mm^3^ sensor consists of four piezoresistors implanted at the roots of a cross-shaped structure measuring the displacement of the elevated pin (Figure 1D). The MEMS sensors are placed on a rigid-flex printed circuit board lodged in the fingertip (see Figure 1C). The resulting array has a density of 72 units/cm^2^ (i.e., 16 channels/22.3 mm^2^), similar to the 70 units/cm^2^ of human Merkel mechanoreceptors (Johansson and Vallbo, 1979).

The piezoresistor output signals are directly (without preamplification) acquired at a frequency of 380 Hz by a 16-channel 24-bit analog-to-digital converter (ADS1258, Texas Instruments) lodged in the distal phalanx. The digital signals acquired from the sensor array via the analog-to-digital converter are encoded as ethernet packets by C/C++ software routines running on a soft-core processor (Nios II, Altera) instantiated onboard a FPGA (Cyclone II, Altera), and broadcasted over an ethernet connection.

The outer packaging layer of the fingertip (Figure 1C) is made of synthetic compliant material (DragonSkin, Smooth-On) and has a surface with fingerprints mimicking the human fingerpad (i.e., 400 μm between-ridge distance; fingerprint curvature radius of 4.8 mm in the center of the sensor array; artificial epidermal ridge-height of 170 μm; total packaging thickness of 770 μm; Oddo et al., 2011a).

#### 2.2.3. Platform

The robotic finger, control modules, and processing hardware are mounted on a platform together with a housing for replaceable surface samples (see Figure 1A). Five different surfaces are used in the tactile skill learning experiments (see Figure 1B): two regular-grated plastic blocks with grating-spacings of 320 and 480 μm (labeled ‘grating 320’ and ‘grating 480’, respectively), a paper surface (labeled ‘paper’), and two different denim textiles (labeled ‘fine textile’ and ‘coarse textile’).

The robotic finger and MEMS sensor are handled by separate control and readout modules. To achieve synchronization between finger movements and tactile sensory readouts, we implemented a real-time, combined sensory-motor driver in Java and .NET, which can be easily interfaced from other programming languages.

## 3. Results

### 3.1. Example: restricted chain walk

#### 3.1.1. Setup

**Figure 3:**
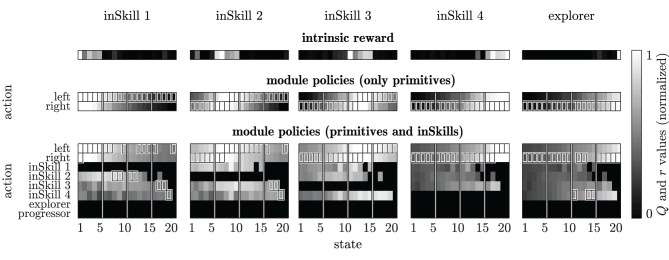
**Intrinsic rewards and module policies after 200 learning episodes in the restricted chain walk environment. Top row:** normalized intrinsic reward for each module as a function of the reinforcement learning state *s*′. **Middle row:** *Q*-tables for modules that can select only primitive actions, with *Q*-values (grayscale) maximum values (boxes) and the abstractor's cluster boundaries (vertical lines). **Bottom row:** *Q*-tables for modules that can select both primitive actions and inSkills. Black areas in the *Q*-tables indicate state-action pairs that were never sampled during learning. Each column of plots shows the results for an individual module.

We illustrate the relevant aspects of the curiosity-driven learning algorithm with a chain walk problem, an often-used toy-problem in reinforcement learning (e.g., Sutton and Barto, 1998). In the chain walk problem considered here, the learning agent is placed in a simulated environment in which it can move left or right between 20 adjacent states. Going left (right) at the left (right) end of the chain leaves the agent in the same state. The structure of the environment is rather obvious when presented in the manner of Figure 3; however, note that the agent does not know beforehand which actions lead to which states. Instead, it has to *learn* the effects of its actions by trying the actions one at a time.

Learning is done over a number of episodes in which the agent always starts in state 1 (left of each column in Figure 3), and interacts with the environment for a maximum of 25 timesteps. Limiting the chain walk task in this fashion forces the learning agent to address the three machine learning challenges discussed in section 2.1.2: (1) the agent can collect only a limited number of samples before it is sent back to state 1; (2) states cannot be equally sampled, as the agent needs to pass through states closer to state 1 to reach more distant states; (3) larger parts of the input space become available to both the RL and the abstractor as a result of learning, requiring the adjustment of the modules to the increasing input space.

In externally-rewarded chain walk tasks, reaching a particular state or states usually yields a reward. Here, however, we let the agent first explore the chain walk environment without providing any external reward. During this curiosity-driven exploration phase, the sensory input to both the RL and the abstractor is the current state. The abstractor divides the states seen thus far into a number of regions, and the RL modules have to learn policies for reaching each of those regions. In curious-exploration mode the agent thus learns skills for reaching different parts of the environment. These skills can later be used in externally-rewarded tasks where the agent is rewarded for reaching particular states. Instead of retrying all primitive actions starting from state 1 each episode, the agent can then use the learned skills to quickly reach different regions in the environment.

In real-world experiments many processes are going on that affect the learning agent to a certain extent, but cannot all be explicitly represented. The effect of these processes is often referred to as noise. To test the robustness of the learning agent against such noise, we incorporate some random processes in both the environment and the abstractor. To simulate noise in the environment, there is a 10% chance that a primitive action has the reverse effect (going right instead of left, and v.v.), and an additional 10% chance that a primitive has no effect at all (the agent stays in the same state). Abstractor noise is introduced by feeding a randomly selected state (instead of the current state) as input to the abstractor with a 10% chance every timestep.

The abstractor used for skill learning is a simple clustering algorithm that equally divides the states seen thus far into *k* parts *y*_1_,…,*y*_*k*_. The *k* corresponding inSkill modules ${ℳ}_{j},\ldots ,{ℳ}_{k}$ learn policies for reaching each of those parts. Rapid exploration of the environment is facilitated by an explorer module. The overall behavior of the agent is driven by a progressor module, which receives reward for the long-term inSkill change and a reward of *r*_*x*_ = 0.1 for selecting the explorer module. In this fashion, the progressor switches to the explorer module once the stability progress of the inSkills becomes smaller than 0.1. Each episode starts with the execution of the progressor in state 1. The progressor selects to execute a module, which runs until it terminates by itself, or for a maximum of τ_*z*_ steps in the environment. This is repeated until τ_*e*_ environment steps (episode length) are executed. At the end of an episode, the samples collected during that episode are used to update the Markov model and the abstractor. Next, the new reinforcement learning policies are generated for each module from the model. A list of all parameter values used for this experiment is given in Table 1.

**Table 1:** **Experimental parameters and their values**.

**Symbol**	**Description**	**Chain walk**	**Tactile platform**
S	state set	{1,…,20}	{1,…,36}, see Figure 8
A	action set	{left, right}	see Figure 8
*k*	number of clusters/inSkill modules	{4, 7, 10}	5
∈	reinforcement learning exploration rate	0.1	0.1
γ	reinforcement learning discount	0.95	0.95
κ	LSPI feature vector length	20	36
*r*_*x*_	fixed exploration reward	0.1	0.2
τ_*e*_	episode length	25	25
τ_*z*_	maximum module timesteps	25	20
*Markov model update weights*			
*w*_*d*_	duration update weight	0.2	0.2
*w*_*f*_	action failure penalty	0.1	0
*w*_*o*_	pruning threshold	0.01	0.01
*w*_*p*_	transition probability update weight	0.33	0.33
*w*_*q*_	long-term reward update weight	0.2	0.2
*w*_*r*_	short-term reward update weight	0.2	0.2
*w*_*z*_	termination probability update weight	0.2	0.2

#### 3.1.2. Skill learning

**Figure 4:**
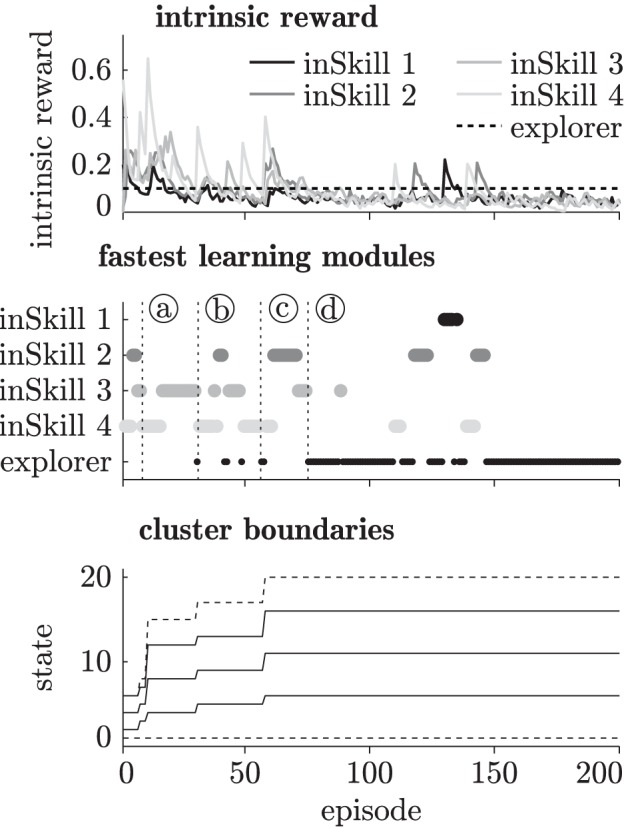
**Modular intrinsic reward (top), fastest learning modules (middle) and the abstractor's cluster boundaries (bottom) during 200 learning episodes in the chain walk environment.** The vertical dotted lines at markers (a–d) indicate distinctive learning events as explained in section 3.1.2.

Figure 4 shows an example of the intrinsic reward, the fastest learning modules, and the changing cluster boundaries of the abstractor during curious exploration with four inSkills. As becomes clear from this figure, the agent starts by learning policies for reaching the first few abstractor states (episodes 1–10, until marker (a)). Once it reaches state 15 at marker (a), the agent spends several episodes (11–30, marker (a)–(b)) adjusting modules 3 and 4, which are the modules that take the agent to the rightmost part of the known environment. At episode 30 (marker (b)), the inSkill modules have stabilized (i.e., all inSkills' intrinsic rewards <0.1), and the agent executes the exploration module. Using the learned skills for further exploration of the environment, the exploration module quickly manages to reach state 17 (episode 31, marker (b)). The abstractor adjusts its cluster distribution to the new observations, and the inSkills have to change their policies for reaching those clusters accordingly. This process is repeated at episode 57 (marker (c)), where the exploration policy is selected, and promptly takes the agent to the rightmost state (state 20). Due to the change in the abstractor's distribution, the inSkills change their policies again until their learning progress becomes less than 0.1 (episode 75, marker (d)), and the exploring module takes over. This switching between learning stable behaviors, and exploiting the stabilized behaviors to explore all transitions in the environment goes on until the environment is fully explored. After that, the agent continues to explore while the inSkills remain stable, indicating that the limit of the agent's learning capabilities in the environment is reached.

An example of the final RL policies for the four-inSkill chain walk task is plotted in Figure 3. The top row of this figure shows an equal distribution of the inSkill reward regions over the state space. The second row with the module's policies in case only primitive actions are allowed, makes clear that the inSkills correctly learn to go left (right) when they are to the right (left) of the reward regions. The bottom row of Figure 3 shows the module's policies in case both primitives and inSkills could be selected as actions. Note that an inSkill cannot execute itself, the explorer or the progressor as part of its policy, as indicated by the absence of *Q*-values in Figure 3. During initial exploration, the selection of other modules happens quite often, because the transition probabilities of primitive actions are not yet sampled reliably. Once the transition probabilities are estimated more accurately during subsequent exploration, primitive actions become preferred over executing other modules, because the on-policy ∈-greedy behavior of the executed modules is less efficient than the optimal policy. After the whole environment is explored, some modules still select other inSkills in certain states. For example, inSkill 1 selects inSkill 2 for going left in states 8–9 and 11–12, and inSkills 1 and 2 select inSkill 3 for going left in states 17 and 18. Again, this is due to the low number of times those state-action (state-module) pairs are sampled during the 200 learning episodes.

The explorer module (right column in Figure 3) learns policies for reaching the least-visited parts of the environment. This is still reflected in the exploring module's *Q*-values and reward after learning. More reward is obtained in states further away from the starting state 1, and *Q*-values are increasing with increasing distance from the starting state, because states further away from the starting state are visited less frequently.

#### 3.1.3. Skill exploitation

To demonstrate the usefulness of the learned skills in an externally-rewarded chain walk task, we compare an agent with trained inSkills against two other learning agents that have no skill-learning capabilities (1) an agent with no additional modules and (2) an agent with a naive explorer module only. Note that all agents still use an ∈-greedy policy as additional means of exploration. The externally-rewarded task for the agents is to reach any of the states in the furthest region (states 16–20), while starting from state 1. The main challenge is getting to this region by fast and efficient exploration. Once the reward region is reached for the first time, the RLs can usually extract the right policy instantly from the model. Each episode lasts only 25 timesteps, and each module can also run for a maximum of 25 timesteps (see Table 1). Together with the 20% chance of primitive failure (10% in the opposite direction and 10% no change) this makes the task particularly challenging. Even when the right policy is learned, the RL might not always reach any of the goal states during an episode due to action noise.

**Figure 5:**
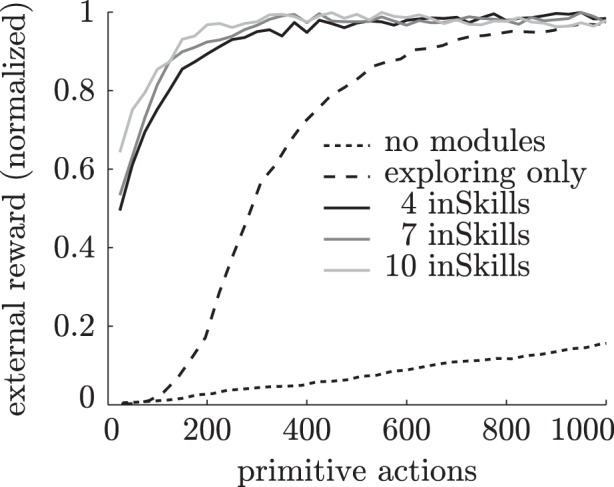
**Normalized external reward obtained by different learning agents during training over 40 episodes (1000 primitive actions) in the chain walk task**.

All experiments are repeated 500 times, and the results are averaged. Figure 5 shows the average proportion of the total possible reward achieved as a function of the number of primitive actions taken during learning over 40 episodes. As becomes clear from this figure, the agent with no additional modules takes a long time to reach the target region. Eventually, the ∈-greedy policy will take this agent to the rewarding states, but on average it takes much longer than the 40 training episodes displayed here. The agent with the explorer module learns to reach the rightmost region much faster, because its explorer module drives it to previously unexplored regions. The agents with previously learned inSkills quickly reach the target region by simply selecting one of the previously learned skills that leads there. Agents with more inSkills collect the reward with less training examples because several modules lead to the rewarding region. Due to the difficulty of the task (20% action failure, 10% abstractor noise), it still takes these agents some episodes to reach the target region for the first time (e.g., less than half of the time for the four-inSkill agent during the first episode). However, the agents with previously-learned skills are still much faster in solving the externally-rewarded task than the other agents.

### 3.2. Curiosity-driven skill learning on the robotic platform

#### 3.2.1. Setup

The curiosity-driven learning algorithm is applied on the robotic tactile platform to learn the movements that lead to different kinds of tactile events. Here, tactile events are encoded as the frequency spectra of MEMS sensor-readouts during 0.33 s finger movements. We filter the MEMS signals with a high-pass filter with lower limit of 0.5 Hz because frequencies below this threshold do not reflect any information about the type (or presence) of sensor-surface contact. Additionally, we filter the signals with a 50 Hz notch filter to suppress power line noise. For various reasons (e.g., location relative to the fingerprints, DragonSkin becoming stuck inside the sensor after intensive use, general wear, 50 Hz distortions), some channels of the MEMS sensor gave less consistent readings than others. The spectra of the three best performing channels selected from visual inspection of the signals are used in the following.

**Figure 6:**
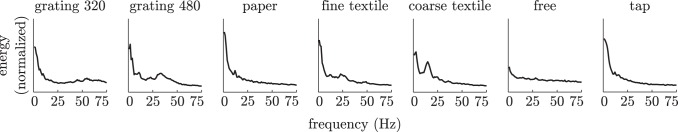
**Average frequency spectra of MEMS recordings during 0.33 s free, tap and slide finger movements for five different surfaces**.

We expect that at least three different tactile events can be distinguished with the robotic platform: (1) movement without sensor-surface contact, which we call free movement, (2) tapping on a surface, and (3) sliding over a surface. To check our expectations, we programmed the finger to perform 50 repetitions of each of these movements in setups with five different surfaces. Figure 6 shows the frequency spectra of the MEMS signals averaged over 50 scripted free, tapping and sliding movements over the surfaces. Sliding movements generate spectra with a low-frequency peak caused by changes in pressure during sliding, and some additional spectral features at higher frequencies: grating 320 has a slight increase in energy around 55 Hz, grating 480 has a peak around 30 Hz, paper has no additional spectral features, fine textile has a peak around 25 Hz, and coarse textile has a peak around 12 Hz. Movements without sensor surface contact (free) yield an almost flat frequency spectrum, while tapping movements lead to spectra with a low-frequency peak and no other significant spectral features.

**Figure 7:**
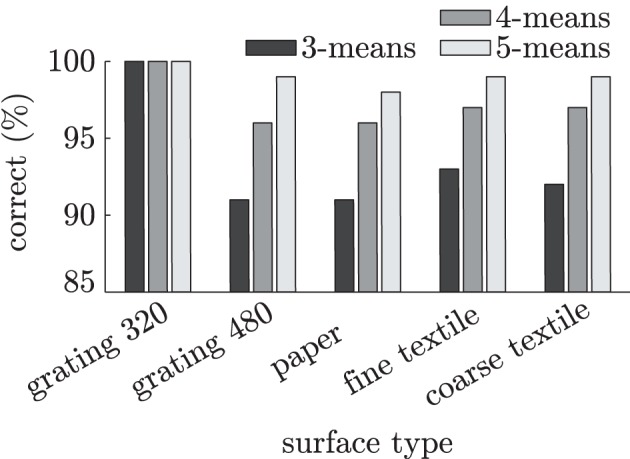
**Clustering accuracies on MEMS frequency spectra during 0.33 s free, tap and slide finger movements for five different surfaces**.

The frequency spectra are fed to an abstractor, whose task is to cluster similar sensory events and represent them as discrete tactile states. The abstractor used for distinguishing tactile events is a *k*-means clustering algorithm (Lloyd, 1982) that partitions the spectra into *k* clusters *y*_1_,…,*y*_*k*_, with *k* ∈ {3, 4, 5}. Although *k*-means clustering is an unsupervised method, it is still possible to calculate its classification accuracy on free, tapping and sliding events by assigning each cluster to the tactile event with the largest number of samples in that cluster. Figure 7 shows the classification accuracies on free, tapping and sliding events for each surface individually. As becomes clear from this figure, the signals generated during free, tapping and sliding movements can be distinguished from each other by the *k*-means clusterers with reasonable accuracy (>90%). Using more than three (i.e., the number of different finger movements) clusters helps to better separate the different tactile events, usually because the difference between data generated during different types of sliding movements is larger than the difference between data collected from free and tapping movements.

**Figure 8:**
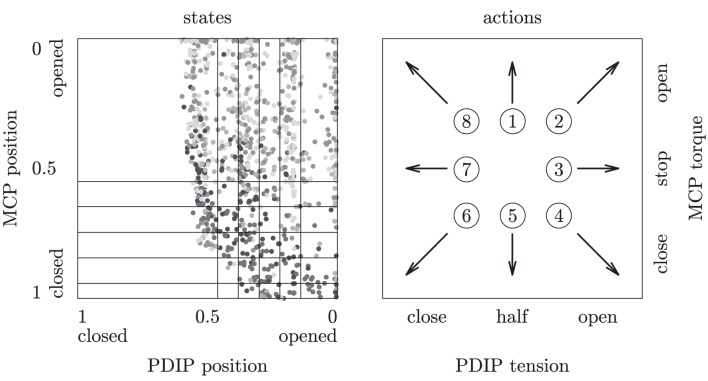
**States and actions of the robotic finger during the reinforcement learning tasks.** Left: 6 × 6 areas in normalized encoder-position space represent the discrete reinforcement learning states. One thousand continuous encoder values (gray dots) obtained from a random policy indicate the finger's movement range in the state space. Right: eight motor actions set the PDIP tension and MCP torque.

The goal of the inSkill modules is to learn behaviors that produce MEMS signals belonging to the corresponding abstractor cluster. To learn finger behaviors, the RLs need some proprioceptive information from the finger, and needs to be able to execute finger movements. We use a representation that might not be optimal for the learning algorithms, but greatly simplifies the graphical presentation of the tactile skill learning. The RLs are fed with the MCP and combined PDIP motor locations, discretized into six positions for each motor, yielding 36 states in total as depicted in Figure 8. The RLs can select from a total of eight primitive actions that set the torque of the MCP and the tension of the PDIP as presented in Figure 8. Each motor primitive lasts a fixed 0.33 s. Unlike the chain walk task, the adjacent states might not be directly reachable from each other, for example, closing the PDIP motor when it is half closed (third or fourth state column in Figure 8) might fully close it at the end of the transition (left state column in Figure 8). Note that many aspects of the robot's dynamics, such as the angles of the underactuated PIP and DIP joints, the precise encoder values, finger movement direction and velocity, cable tension in case of sensor-surface contact, etc., are not captured in this representation, and instead need to be absorbed by Markov model's transition probabilities. More complex representations using more state and action dimensions might facilitate faster learning, but do not lend themselves for an easily understandable presentation of tactile-skill learning.

The episodic learning scheme in the chain walk task is also applied for the robotic platform. At the episode start the finger is put in randomly selected encoder positions in the range (0.1–0.9) (MCP) and (0.1–0.5) (PDIP), which approximately covers the finger's movement range (see Figure 8). We use slightly shorter episode and module runtime lengths (20) than in the chain walk task to speed up the experiments. A list of all parameter values used for the robotic platform experiment is given in Table 1.

We run the curiosity-driven learning agent on the robotic platform using five inSkill modules, a naive explorer and a skill progressor. No external reward is provided to the agent yet. To allow for adaptation of the *k*-means clusterer during exploration and skill-learning, it is retrained every reinforcement learning episode on a buffer of the last 500 observations. Consistency of the cluster-means between each episode is enforced by initializing the *k*-means training algorithm with the most recent cluster-means.

#### 3.2.2. Skill learning

**Figure 9:**
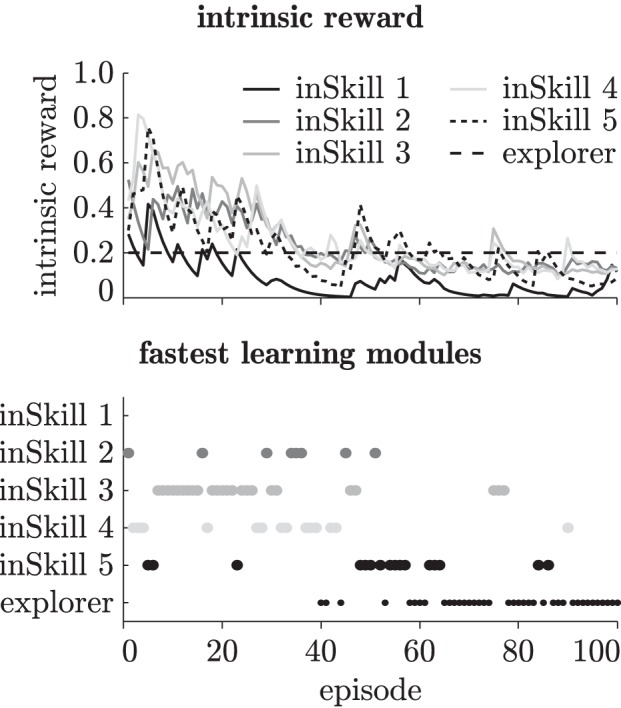
**Intrinsic reward (top) and fastest learning modules (bottom) during 100 learning episodes on the robotic platform with the coarse textile**.

Figure 9 shows an example of the intrinsic reward of the inSkills during curious exploration of the robotic platform with the coarse textile. The intrinsic reward generated by the progress of the inSkills decreases over time as the agent learns separate behaviors for generating different tactile events. Unlike in the chainwalk task, little switching back and forth between skill learning and exploration occurs. Instead, the agent learns the inSkills without calling the explorer for explicit exploration, because it can easily reach all parts of the environment. After about 65 episodes, the inSkills stabilize, and the exploring modules takes over, with a few short exceptions around episodes 75, 85, and 90. The learning progress in the inSkills around these episodes is due to the finger getting stuck (caused by faulty encoder readouts) in a pose where the sensors generated many samples for a single cluster. The curiosity-driven learning algorithm picks this up as a potentially interesting event, and tries to learn behaviors that reliably lead to such an event. However, after resetting the finger at the end of the episode, the encoders return the correct values again, and the learning agent gradually forgets about the deviating event.

**Figure 10:**
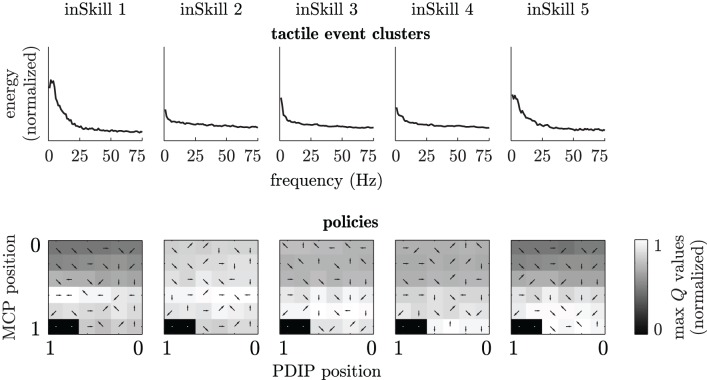
**InSkill sensory clusters and policies after 100 learning episodes in a setup with the coarse textile.** Top row: frequency-spectra cluster means of each inSkill. Bottom row: normalized maximum *Q*-values for each module (grayscale) and best actions (arrows). States and actions are the same as in Figure 8. Black areas without arrows indicate states that were never sampled during learning.

Figure 10 shows an example of the abstractor clusters and corresponding RL policies after 100 episodes of curiosity-driven learning in a setup with the coarse textile. Comparing the cluster-means of the inSkills learned during curious exploration to the frequency spectra of the scripted free, tapping and sliding movement in Figure 6, it becomes clear that the abstractor has learned a similar division of the MEMS frequency spectra. The almost flat frequency spectrum for inSkill 2 is very similar to the frequency spectrum of the scripted free movements, and the spectrum for inSkill 1 has a similar low-frequency peak as the spectrum of the scripted tapping movement. The spectrum of inSkill 5 is most similar to the sliding spectrum of the coarse textile in Figure 6, but misses the characteristic peak around 12 Hz. The absence of a clear peak for this inSkill is probably due to the combination of several sliding movements at different sensor angles and hence, different sensor-surface speeds, smearing out the spectral peaks over a larger range. The actual behaviors generated by these inSkills and the *Q*-tables in Figure 10 indicate that the corresponding finger movements are also learned; the *Q*-values of inSkill 2 (free) have almost the same value throughout the state space, with slightly higher values with the finger away from the surface; inSkill 1 (tap) has two distinctive high *Q*-values for opening and closing the PDIP joints with the MCP joint halfway closed (middle-left in its *Q*-table); inSkill 5 (slide) obtains high *Q*-values with the MCP joint almost closed and opening and closing actions close to the surface (bottom-center in its *Q*-table).

The specialization of inSkills 3 and 4 is less obvious from Figure 10. However, the actual behavior of the inSkills indicated that inSkill 3 developed into a module for learning slight elastic deformations of the sensor packaging material while opening the finger close to the surface (while not actually touching anything), while inSkill 4 developed behavior that led to similar changes during closing movements.

In setups with the other four surfaces, the skill repertoire learned by the agent also contains distinct behaviors for free, tapping and sliding movements. Apart from these skills, a range of other consistent behaviors were learned, such as behaviors for breaking sensor-surface contact, behaviors that generate elastic deformation of the packaging material after sensor-surface contact, separate skills for sliding forward and sliding backward, hard and soft tapping, and tapping from different angles.

While the specialization of the skills changes during exploration of the environment, the exploration phase often involves the learning of skills in a particular order; first the agent learns to distinguish between free and tapping movements, and as a result of improving its tapping skills, learns a sliding skill as well. During the learning of reliable tapping, the finger makes many movements with the sensor close to the surface. This leads to the discovery of sliding movements, and the learning of the associated sliding skill. The result of this sequence is visible in Figure 9, where the sliding skill (inSkill 5) is the last module that is learned (note that the final specialization happens for the inSkill with the highest number (5) is a coincidence; the order of the clusters is determined randomly).

#### 3.2.3. Skill exploitation

After autonomous learning of skills on the robotic platform, we test the usefulness of the learned skills in an externally-rewarded surface-classification task. The task for the robotic finger is to figure out which surface sample is placed on the platform. Instead of programming the finger to slide over the surface, the agent has to learn which of its movements generate the most distinguishing information about the surface sample. We compare the learning agent with previously learned tapping and sliding skills against a learning agent without such previously-learned skills.

To determine the different surface types in the externally-rewarded task, we compare the frequency spectra recorded during each finger movement with previously recorded frequency spectra during sliding movements over the different surfaces, tapping movements, and movements without sensor-surface contact. An external reward of 1 is provided when the recorded spectrum closely matched the frequency spectra of the surface placed on the platform and an external reward of 0 otherwise. After each correct classification, the module ends, and the finger is reset as in an episode start. Although the reward function does not directly represent misclassifications (i.e., the finger can continuously provide misclassification without penalty) due to the limited amount of time in each trial, more reward can be obtained if the finger makes correct classifications sooner.

**Figure 11:**
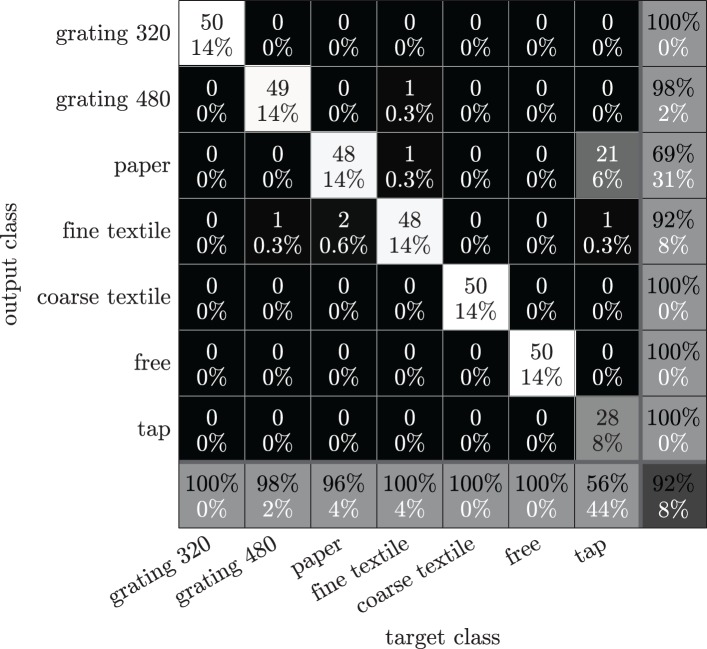
**Confusion matrix for surface-type classification using a 10-means clusterer on frequency spectra recorded during pre-scripted free, tapping and sliding movements.** Background colors indicate the number of samples assigned to each class. The number and percentage of correctly (incorrectly) classified samples are indicated in black (white) text.

To give an indication of how difficult it is to distinguish the different surfaces during scripted sliding movements, we provide the frequency spectra recorded during sliding movements as well as during free and tapping movements to a 10-means clusterer, and compute the clustering accuracy as described before. As shown in Figure 11, the overall accuracy of 92% for distinguishing the different surfaces from each other, is not as high as the accuracy of distinguishing between free, tap and slide movements for each surface individually (Figure 7), but still is well above guess chance (14%). Figure 11 further indicates that sliding movements over different surfaces can be accurately distinguished from each other, as well as from tapping and free movements. However, it is more difficult to distinguish sliding movements over paper from tapping movements, probably because the smooth paper surface produces almost no distinctive spectral features (compare also the frequency spectra for sliding over paper and tapping in Figure 7). Using slightly different numbers of clusters (between 7 and 15) changed the accuracies with only a few percentages.

During the externally-rewarded task, we keep training the skills learned during the curious exploration phase for two reasons: (1) skills used during autonomous exploration might be useful in quickly solving an externally-specified task, but might not solve it directly. For example, a sliding skill might lead to sensor data that distinguishes a sliding movement from a tapping movement in a particular setup, but might not necessarily be good for distinguishing between different surface types. Still, some kind of sliding movement is probably required to distinguish between different surface types. An existing sliding skill could be easily adjusted to make slightly different movements that are better for distinguishing between surface types. We therefore add the external reward to inSkill modules that were active when the external reward was received, and adjust the modules' models and policies accordingly. (2) The dynamics of the robotic platform change during operation for various reasons (e.g., cable stretch, changes in ambient temperature and battery levels, general wear). While this might require repeated calibration in traditional approaches, the learning system used here is flexible enough to cope with those changes.

**Figure 12:**
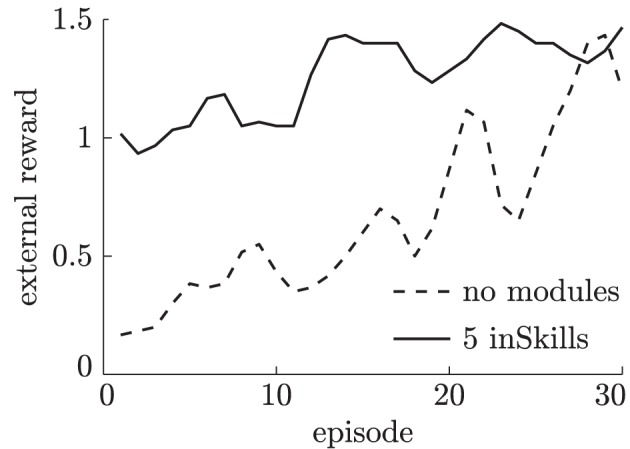
**External reward obtained by different learning agents during training over 30 episodes in the surface-classification task**.

Learning in the externally-rewarded tasks is done over 30 episodes and repeated three times for each of the five surfaces described in section 2.2.3. Figure 12 shows the average reward during training. As shown in this figure, agents that have previously learned inSkills learn to solve the externally-rewarded task much faster than the agent without such previously learned skills. The skills learned during the curious exploration phase are useful for the externally-rewarded task, but often do not solve it directly. Instead, the skills need to be (slightly) adjusted from skills that distinguish free, tap and slide movements for individual surfaces, into sliding movements that distinguish different surfaces. This skill adjustment is reflected in the increasing reward of the agent with previously-learned inSkills while it is learning to solve the externally-rewarded task.

## 4. Discussion

We presented a curiosity-driven modular reinforcement learning framework for autonomous learning of tactile skills on a tactile robotic platform. The learning algorithm was able to differentiate distinct *tactile events*, while simultaneously learning *behaviors* for generating those tactile events. The tactile *skills* learned in this fashion allowed for rapid learning of an externally-specified surface classification task. Our results highlight two important aspects of active tactile sensing: (1) exploratory tactile skills can be *learned* through intrinsic motivation (2) using previously-acquired tactile skills, an agent can learn which exploratory policies yield the most relevant tactile information about a presented surface.

A key aspect of the developmental learning system presented here is the ability to use previously-learned skills for reaching novel parts of the environment, and to combine skills into more complex composed skills. This bootstrapping of skills became apparent in the chain walk task, where modules used other modules to reach parts of the environment in case the transition probabilities of primitive actions were not accurately known. Also, during curious exploration of the tactile platform, the agent first learned to move the finger without sensor-surface contact, then learned to tap the finger on the surface, and finally learned the more difficult skill of sliding the finger over the surface while maintaining sensor-surface contact. After learning these skills, the agents kept exploring the environment in search for further things to learn, while maintaining a stable division of skills learned thus far.

The notion of *active* tactile sensing has recently been discussed in Prescott et al. (2011), who considered different interpretations: (1) the energetic interpretation, in which the information-relevant energy flow is from the sensor to the outer world being sensed; (2) the kinetic interpretation, in which the sensor touches rather than is being touched; and (3) the preferred interpretation by Prescott et al. (2011), which considers active sensing systems as purposive and information-seeking, involving control of the sensor apparatus in whatever manner suits the task. Our work fits best with the third interpretation, because tactile information drives both the learning of tactile exploratory skills and the categorization of tactile stimuli. Note, however, that the exploratory dynamics are not directly used as kinaesthetic information provided to the texture classifier, but rather enter in the categorization chain as affecting sensor outputs. In future work, it would be interesting to study if and how tactile and kinaesthetic information could be fused for motor control and perceptual purposes during learning of exploratory skills.

A potentially interesting comparison could be made between the usage of sensory information during tactile exploration in humans and in the biomimetic robotic setup. In our experiments the algorithms were able to distinguish between different textures using the key spectral features of the sensor output. The human neuronal mechanisms and contributions of the different types of mechanoreceptors for distinguishing textural details (Yoshioka et al., 2007) are still highly debated. No agreement has been reached about the most informative mechanoreceptors (i.e., among Merkel, Meissner, Ruffini, and Pacini corpuscles) or about the coding strategy (e.g., temporal, spatial, spatiotemporal, intensity) used by humans to represent textural information. Various studies aimed at demonstrating that the Pacinian system encodes fine textures (Hollins et al., 2001; Bensmaïa and Hollins, 2003; Bensmaia and Hollins, 2005). In particular, Hollins and Risner (2000) supported the Katz's duplex theory, according to which fine textures are supposed to be mediated by different classes of mechanoreceptors via vibrational cues for fine forms and via spatial cues for coarse forms. Conversely, Johnson and colleagues presented human psychophysical studies and complementary electrophysiological results with monkeys supporting a unified peripheral neural mechanism for roughness encoding of both coarse and fine stimuli, based on the spatial variation in the firing rate of Slowly Adapting type I afferents (SAI; Merkel) (Connor et al., 1990; Connor and Johnson, 1992; Blake et al., 1997; Yoshioka et al., 2001). Johansson and Flanagan (2009) introduced a hypothetical model of tactile coding based on coincidence detection of neural events, which may describe the neuronal mechanism along the human somatosensory chain from tactile receptors, passing through cuneate neurons up to the somatosensory cortex. What has been agreed on is that humans can detect up to microtextures (LaMotte and Srinivasan, 1991), and that the human perception of textures is severely degraded in case of lack of tangential motion between the fingertip and the tactile stimuli (Morley et al., 1983; Gardner and Palmer, 1989; Radwin et al., 1993; Jones and Lederman, 2006). This consolidated finding fits well the results presented in the current work: like human beings, the robotic finger also developed skills for sliding motions tangential to the tactile stimuli while seeking for information-rich experiences.

Recently, Fishel and Loeb (2012) obtained impressive texture classification accuracies on a large range of different textures, using an algorithm that selects the most discriminative exploratory motions from a set of tangential sliding movements with different forces and velocities. *That* variations in high-level motion parameters like force and velocity are important for obtaining the most distinctive information is, however, not inferred by their learning algorithm. Our approach first learns how to make exploratory movements without any teacher feedback and without any knowledge of high-level parameters such as sensor-surface force and velocity. As in Fishel and Loeb (2012), our algorithm then learns to select exploratory movements that yield the most distinctive information about the presented textures. Whereas Fishel and Loeb (2012) learn to select pre-scripted exploratory movements, our algorithm can still refine the previously-learned exploratory movements during the learning of the supervised texture classification task.

A further comparison could be made between the exploratory behaviors learned by the biomimetic platform, and the learning of tactile exploratory procedures by human beings. There is a large body of literature about the exploratory procedures employed by humans when investigating an objects, including texture (e.g., Lederman and Klatzky, 2009, and references therein). Tapping and sliding tangentially over a surface are both used by human beings and learned by the robotic platform when gathering tactile information. Apart from using or selecting *existing* exploratory procedures it could also be interesting to study similarities in how these exploratory procedures are *learned* in human beings in the first place. The constraints of the biomimetic robotic finger make tapping easier to learn than sliding. Consequently, sliding is often learned after and as a result of tapping. Similar constraints in human beings might lead the same developmental trend (from tapping to sliding).

Although the robotic finger has just two controllable degrees of freedom, learning skills in an autonomous fashion already proved to be beneficial during learning of an additional externally-specified task. Moreover, the learning approach allowed for overcoming challenges in traditional engineered solutions to robotic control, such as the need of constant recalibration of the robotic platform to changing circumstances, and the absence of joint-angle sensors in the underactuated joints. We expect that autonomous acquisition of skills in robots will become increasingly important for autonomous learning in robots with more degrees of freedom and sensory capabilities.

### Conflict of interest statement

The authors declare that the research was conducted in the absence of any commercial or financial relationships that could be construed as a potential conflict of interest.

## Glossary of symbols.

A: action set
*A*: LSPI update matrix
*a*: action
*b*: LSPI update vector
*c*: transition count in Markov model
*D*: set of samples
*d*: transition duration
*e*: base of natural logarithm
*i*: index
*j*: module index
*k*: number of clusters / inSkill modules
M: reinforcement learning module
*m*: Markov model value
*n*: number of samples
*o*: Markov model pruning threshold
*Q*: reinforcement learning state-action value
*p*: transition probability
*q*: long-term reward
*r*: short-term reward
*s*, *s*′: state
S: state set
*t*: time
*v*: observed value
*w*: Markov model update weight
*y*: abstractor output
*z*: termination probability
Δ: difference operator
∈: reinforcement learning exploration rate
γ: reinforcement learning discount
κ: LSPI feature vector length
π: reinforcement learning policy
ɸ: LSPI feature vector
ω: LSPI weight
τ_*e*_: episode length
τ_*z*_: maximum module timesteps

**Glossary of acronyms.**
DC: direct current
DIP: distal interphalangeal
exSkill: externally-rewarded skill
inSkill: intrinsically-motivated skill
MCP: metacarpophalangeal
MEMS: microelectromechanical system
PDIP: combined PIP and DIP joints
PIP: proximal interphalangeal
RL: reinforcement learner
